# Identification of care tasks for the use of wearable transfer support robots – an observational study at nursing facilities using robots on a daily basis

**DOI:** 10.1186/s12913-021-06639-2

**Published:** 2021-07-05

**Authors:** Kenji Kato, Tatsuya Yoshimi, Shohei Tsuchimoto, Nobuaki Mizuguchi, Keita Aimoto, Naoki Itoh, Izumi Kondo

**Affiliations:** 1 Laboratory for Clinical Evaluation with Robotics, Center of Assistive Robotics and Rehabilitation for Longevity and Good Health, National Center for Geriatrics and Gerontology, 7-430, Morioka, Obu, Aichi 474-8511 Japan; 2 Department of Rehabilitation Medicine, National Center for Geriatrics and Gerontology, 7-430, Morioka, Obu, Aichi 474-8511 Japan; 3 Center of Assistive Robotics and Rehabilitation for Longevity and Good Health, National Center for Geriatrics and Gerontology, 7-430, Morioka, Obu, Aichi 474-8511 Japan

**Keywords:** Wearable transfer support robots, Physical burden, Care task, Nursing facility, Observational time-motion analysis

## Abstract

**Background:**

To reduce the physical burden of caregivers, wearable transfer support robots are highly desirable. Although these robots are reportedly effective for specific tasks in experimental environments, there is little information about their effectiveness at nursing care facilities. The aim of this study was to identify care tasks and operations suitable for the use of these robots among caregivers in nursing facilities where these robots have been in use on a daily basis.

**Methods:**

A 1-min observational time-motion analysis was conducted to examine care tasks and operations in two nursing facilities where wearable transfer support robots, namely Muscle Suit or HAL® Lumbar Type for Care Support, have been used routinely on a daily basis for more than 24 months.

**Results:**

Analysis of the care tasks and their time ratio while wearing the equipment revealed that both robots were used conspicuously for direct care in over 70% of transits, especially during transfer assistance and toileting care. Furthermore, these robots were used intensively in the morning along with wake-up calls to care recipients, where pre-assigned wearers used them as part of their “routine work.”

**Conclusions:**

We found that these wearable transfer support robots enabled effective performance of care tasks and operations in nursing facilities where these robots have been used on a daily basis for an extended period of time. These results may lead to the effective implementation and sustained operation of other types of care robots in the future.

**Trial registration:**

UMIN Clinical Trials Registry no. UMIN000039204. Trial registration date: January 21, 2020. Interventional study. Parallel, non-randomized, single blinded.

**Supplementary Information:**

The online version contains supplementary material available at 10.1186/s12913-021-06639-2.

## Background

**Fig. 1:**
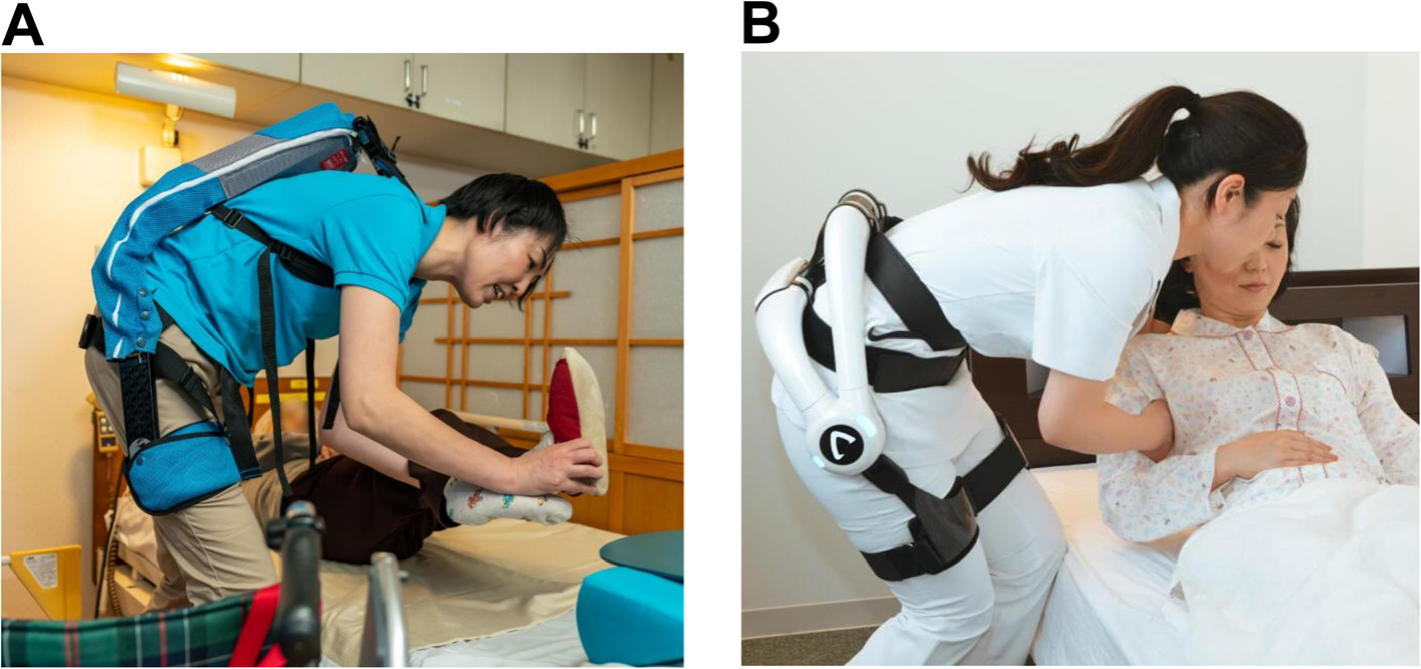
Images of the Muscle Suit (MS), developed by INNOPHYS Co. Ltd. in Japan (**A**), and the HAL®, Lumbar Type for Care Support (HAL), developed by CYBERDYNE Inc. in Japan (**B**) being worn for care work. The images used in this figure were kindly provided by INNOPHYS Co. Ltd. (**A**) and CYBERDYNE Inc. (**B**) with permission for use in publication. The person wearing these robots and the person on the bed are both models and they are not participants of this study

In recent years, the shortage of caregivers due to a super-aging society has resulted in the need to reduce the burden on caregivers and improve the quality of care through the use of robotic care equipment [1]. In particular, the incidence of chronic low back pain among caregivers is increasing [2], and there is a pressing need to reduce their burden from repetitive motions such as transfer assistance and repositioning of care recipients on a bed, which increase the risk of low back pain [3, 4]. In the United States, the use of support equipment, such as lifting equipment, has been recommended as a countermeasure to this issue [5] and has made a significant contribution to the prevention of low back pain among caregivers [6–8]. In Japan, newly developed wearable robotic care equipment for transfer assistance is available as a means of supporting caregivers to reduce their physical burden. For example, the Muscle Suit (MS), developed by INNOPHYS Co. Ltd. in Japan (Fig. 1A), uses pneumatic artificial muscles to support a caregiver’s body by assisting with movements such as lifting/transferring a person or an object and keeping a mid-height posture [9]. HAL®, Lumbar Type for Care Support (HAL), developed by CYBERDYNE Inc. in Japan (Fig. 1B), assists caregivers movement utilizing their bio-signals and supports their lower back to reduce the stress applied to their lumbar region [10]. These robots have been described as “wearable equipment for transfer aids” in the priority area of care robot technology in Japan [11] and are expected to be used in the provision of nursing care services. Although these robots are effective in reducing muscle fatigue and muscle activity in specific movement tasks in experimental environments [12, 13], there is little information on how they could be utilized efficiently at nursing facilities.

According to a recent survey of all health service facilities for the elderly in Japan (*N* = 1347), approximately 60% of the facilities responded that they would like to consider or are considering the introduction of wearable robotic care equipment for transfer aids. However, only 7.6% of facilities have actually introduced them [14]. It is also reported that more than half of these robots are not utilized effectively even after being introduced [14]. The reasons hampering their introduction include their low cost-effectiveness and difficulties in receiving subsidies for such equipment [15]. One of the reasons for their not being used effectively is that the care tasks or care flow in which they can be utilized may not be defined.

For example, the MS and HAL are expected to be used mainly for tasks related to the administration of direct care to care recipients, such as posture changes and transfer assistance from a bed, which are physically demanding tasks for caregivers. However, there is also potential for the use of these robots in a wide range of care scenarios that are associated with a physical burden, such as assisting with bathing and toileting. Thus, there is a need to investigate in the kinds of tasks that the wearable equipment can assist in all care scenarios. In such an exploratory study, it is first necessary to understand the actual working conditions of nursing facilities that use MS and HAL on a daily basis for a long period of time. Therefore, the objective of this study was to examine care tasks and operations in two nursing facilities in which HAL or MS are used on a daily basis for more than 24 months using observational time-motion analysis [16–18]. We believe that this information is important for effective design, implementation, and sustained operation of care robots in the future.

## Methods

### Settings

In this study, two types of wearable transfer support robots, MS and HAL, which are commercially available and widely used in Japan, were selected from the “Robotic Care Devices Portal - Robotic Devices for Nursing Care Project [11]“, a leading site for nursing care robots in Japan. We selected two special nursing homes for the elderly that had used robots on a daily basis for > 24 months [19]. In general, special nursing homes for the elderly in Japan are for people needing long-term care level 3 or higher of the long-term care classification in Japan [20, 21]. Physical abilities in long-term care level 3 generally represents partial or full assistance when standing up, sitting up, standing on one leg, walking, body washing, eating, nail clipping, putting on and taking off pants, moving around, decision making involved in daily life, facial washing, hair dressing, mouth cleaning, urination and defecation, and transfer from/to bed [20, 21]. These two nursing facilities use the “unit care method”, which divides the entire care facility into units and provides long-term care individually. At the units, we selected 2 days (MS-skilled units, 8.5-h day shift) or 3 days (HAL-skilled unit, 9-h day shift) when there were no irregular events such as annual events at the facility or interaction with the local community, with an interval of about 3 weeks as representative days for the time-motion study.

The two MS-skilled units consisted of 18 full-time caregivers (male, 11; female, 7; age, 38 ± 9 years; work experience, 9 ± 6 years) with 59 care recipients and 3 robots. A total of 8 caregivers, 3 of whom were using robots, were working on the 2 days when the time-motion study was conducted. The one HAL-skilled unit consisted of 7 full-time caregivers (male, 3; female, 4; age, 39 ± 15 years; work experience, 8 ± 8 years) with 9 care recipients and 1 robot. A total of 4 caregivers, 2 of whom were using robots, were working on the 3 days when the time-motion study was conducted. Caregivers that participated in this study consisted of certified care workers with a national qualification in the area of nursing care and care helpers. The duties of care workers and care helpers are similar, but certified care workers are in charge of the unit and provide care instructions to the other caregivers. Workers at both facilities consisted of four certified care workers and three care helpers, and the remaining worker at the MS facility was a prosthetist. All methods were carried out in accordance with relevant guidelines and regulations. Study protocols have been reviewed and approved by the Ethics and Conflicts of Interest Committee of the National Center for Geriatrics and Gerontology (acceptance no. 1275) and all participants provided written informed consent.

### Classification of caregivers’ activities (time-motion study)

**Fig. 2:**
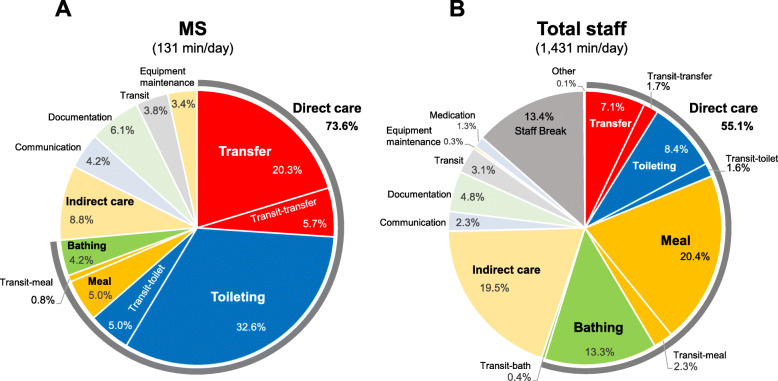
Classification of care services and the time in the unit while wearing the MS (**A**) and the overall caregiving (**B**) were investigated with the observational time-motion analysis

**Fig. 3:**
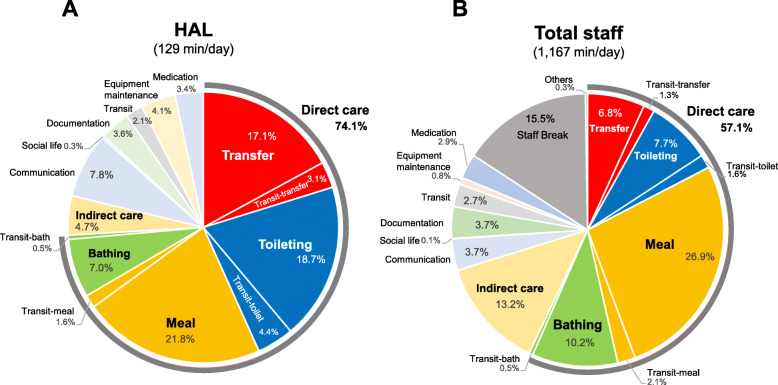
Classification of care services and the time in the unit while wearing the HAL (**A**) and the overall caregiving (**B**) were investigated with the observational time-motion analysis

**Fig. 4:**
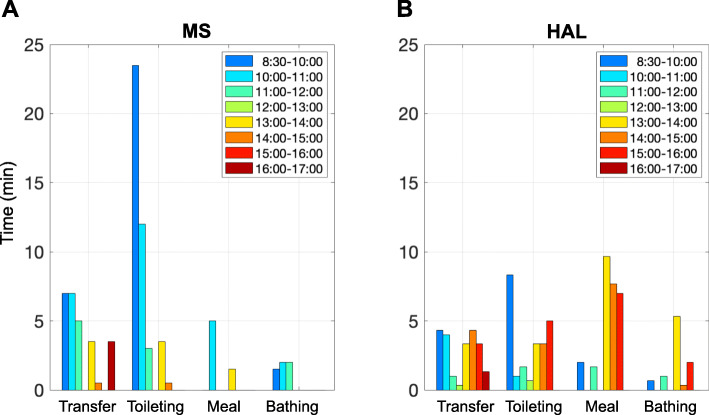
The distribution of robot usage time periods for direct care of transfer, toileting, meal assistance, and bathing assistance in which the MS (**A**) and HAL (**B**) were worn intensively

To identify the care tasks, we used a Japanese version of the 1-min time-motion study (hereafter, time study), similar to one used in an observational time-motion study [16–18]. The time study was conducted as follows: one or two professional observers followed a caregiver throughout their day shift and recorded their behavior and care tasks using codes. The codes were based on a chart produced by the Survey of Elderly Care by the Japanese Ministry of Health, Labour, and Welfare in 2006 [22]. A translation of the chart is shown as Supplementary Table 1. Specifically, “100: Bathing / dressing / changing clothes,” “200: Transfer,” “300: Meal assist,” “400: Toileting,” “500: Washing / cleaning / consultation / waking up and sleeping” (living support for independence),” “600: Events / going out / visitors (social-life support),” “700: Behavioral problems,” “8: Medical care / medication,” “9: Functional training,” “999: Others,” and “0: Care that is not directly related to the recipient,” which contains “contact / recording,” “movement / patrol,” “robot maintenance,” and “hand washing,” are classified. Additionally, the chart is combined with the classification used in a previous study [16] as Direct care, codes 100 to 400; Indirect care, part of codes 500 and 0; Communication, other part of code 500 and code 600; Medical care, codes 800 and 900; Transit, codes 016 and 026; Documentation, codes 012 and 023; Staff break, codes 022 and 024; and Others, codes 700 and 999. This code and whether the staff were wearing a robot or not was classified in the state at 0 s of every minute. Using the data, the tasks and times of all target staff at both facilities were summarized, and those of the staff wearing the robots were also calculated (Figs. 2 and 3). On the basis of the obtained care flow from the time study, we counted the number of transfers and toileting times (Table 1) and particularly the short transits associated with direct care (transit-transfer, transit-toilet, transit-bathing, or transit-meal assist) (Figs. 2 and 3). Lastly, the time of day and usage time of robot use were summarized from the time study (Fig. 4).

**Table 1:** Number of transfers and toileting assistance times with or without the robot by one person per day

		MS			HAL		
		Times/day	%	%	Times/day	%	%
**With the Robot**	**Transfer**	7.5	56.6	44.2	5.3	29.2	45.6
	**Toileting**	5.8	43.4		12.8	70.8	
**Without the Robot**	**Transfer**	11	65.7	55.8	7.0	32.6	54.4
	**Toileting**	5.8	34.3		14.5	67.4	
	**Total**	30			39.5

### Questionnaire and interview for caregivers

**Fig. 5:**
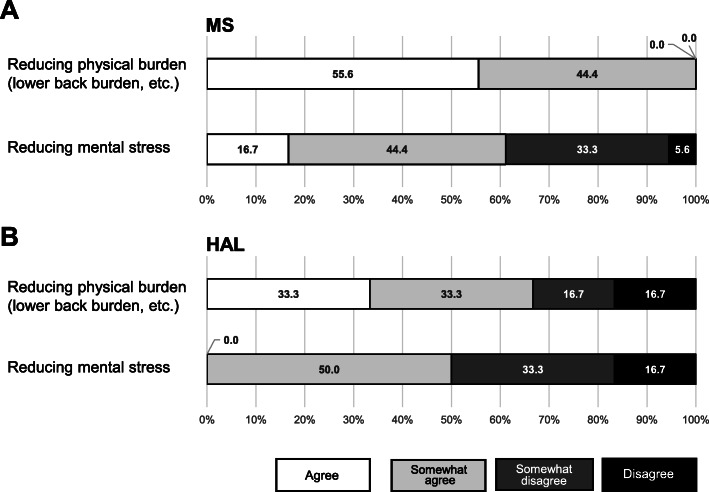
The effects of the MS (**A**) and HAL (**B**) on the physical and mental burdens of the caregivers were investigated using a questionnaire

After the time study, a self-administered questionnaire was used to assess the effects of reducing physical and mental burden on 18 caregivers belonging to the units that used the MS and 7 caregivers that used the HAL (Fig. 5). The answer for each question was selected from “Agree,” “Somewhat agree,” “Somewhat disagree,” and “Disagree.” In addition, based on the results of the survey, we interviewed the staff, including leaders who wore these robots, about the following aspects of the main care tasks: 1) Reasons for using (or not using) robots in each care task, 2) Other ideas in nursing care operations for sustainable use of robots.

## Results

### Identification of care scenarios with the MS by time-motion study

A time study was used to identify the number of caregivers who wore the MS and the time they wore it, as well as the unit’s total work time over 2 days. As a result, the unit’s total work time was 1431 min/day, of which 3 caregivers wore the MS for 131 min/day (Fig. 2). This total time with the MS represented 9% of the unit’s total work time, and 19% of the total work time for the 3 caregivers wearing the MS (690 min/day).

The types of care services and the time in the unit while wearing the MS were investigated with the time-motion study (Fig. 2A). First, care services while wearing the MS were concentrated in “direct care,” with transfer assistance, toileting care, meal care, and bathing care accounting for 73.6% of their total time. In particular, the use of the MS in direct care for transfer assistance (20.3%) and toileting care (32.6%) was notable (Fig. 2A). Conversely, direct care services for transfer assistance (7.1%) and toileting care (8.4%) in overall caregiving were much less than those while using MS, while meal assistance (20.4%) and bathing care (13.3%) were higher than those while using MS. Indirect care accounted for 19.5% of the overall caregiving (Fig. 2B).

As for transits, the time required for a single transit did not exceed 2 min in most cases. Moreover, approximately 70% of transits occurred by direct care for toileting or transfer assistance. Therefore, it is highly probable that the MS was not worn solely for the purpose of transits, but mainly for tasks associated with direct care to the care recipient. As for daily equipment maintenance, the MS requires pumping with air 4–50 times into its artificial muscle at the beginning of use, which may take a few minutes. However, the ratio of time used for equipment maintenances was 3.4% in MS, which is about 5 min a day.

### Identification of care scenarios with the HAL by time-motion study

A time study was also used to identify the number of caregivers who wore the HAL and the time they wore it, as well as the total work time of the unit over 3 days. The total working hours in the unit was 1168 min/day, of which a total of 2 people wore the HAL for 129 min/day (Fig. 3). The time spent wearing the HAL was equivalent to 11% of the total working time of the unit, and for the 2 people wearing the HAL (780 min/day), they wore it for 17% of their total time.

The types of care services and the time in the unit while wearing the HAL were investigated with the time study. Similar to the results for the MS, the HAL was used intensively for direct care (74.1% of total use). However, unlike the MS, it was not only used for transfer assistance (17.1%) and toileting care (18.7%), but also for meal assistance (21.8%). Conversely, looking at the overall work period, direct care accounted for 57.1% of total work time and transfer assistance and toileting care were reduced to 6.8 and 7.7%, respectively, while meal assistance (26.9%) and bathing care (10.2%) were increased. Indirect care also accounted for 13.2% of the total work time. Among these results, it became clear that the HAL usage was concentrated on direct care, but could be utilized for transfer assistance, toileting care, and meal assistance. As a result of interviews with the caregivers, although the HAL was mainly used for care tasks that placed a load on the lower back, such as transfer assistance and toileting care, it was also used for some recipients whom the caregivers needed to maintain a forward leaning posture while sitting. In that case, the HAL was used because the lumbar burden could be reduced.

As for transits, similar to the MS, most of the transits that occurred per trip did not exceed 2 min (less than once a day), and approximately 70% of transits occurred by direct care for toileting or transfer assistance. Maintenance of the HAL consisted mainly of regular daily charging of the battery, which was limited to 4.1% and about 5 min per day.

### The total number of transfers with or without MS/HAL

The total number of transfers made by the caregivers with or without MS/HAL are summarized in Table 1. As a result, the MS was worn for 44.2%of the 30 transfers per caregiver per day that occurred in the overall study period, while the HAL was worn for 34.6% of 39.5 transfers per day. Of the transfer assistance with the robots, 56.6% were for transfers and 43.4% were for toileting care using the MS, while 29.2% were for transfers and 70.8% were for toileting care using the HAL.

### Distribution of robot usage time periods for direct care with MS/HAL

Finally, we investigated the distribution of robot usage time periods for direct care in which the MS or HAL was worn (Fig. 4A). As a result, MS usage was concentrated in the morning, especially for transfer and toileting assistance. In particular, in terms of toileting assistance, the MS was used continuously in the earliest hours of the morning. The use of the HAL robot was concentrated in the morning (Fig. 4B), especially for toileting care. In the case of meal assistance, the proportion of use of the HAL was increased in the afternoon. According to interviews performed after the survey, we confirmed that the use of the MS for intensive toileting and transfer assistance, especially in the morning, was carried out by the pre-assigned MS-wearing caregivers. This was the result of a daily patrol specializing in transfer and toileting assistance around the bed of the recipients that tended to occur in the morning.

### Questionnaire for the caregivers about the MS or HAL

The effects of the MS on the physical and mental burdens of the caregivers were investigated using a questionnaire (Fig. 5A). As a result, all 18 caregivers working in both units using the MS answered “Agree” or “Somewhat agree” to the question “Was physical burden reduced by use of the MS?” Moreover, 61% of them answered “Agree” or “Somewhat agree” to the question “Was mental burden reduced by use of the MS?”. According to the questionnaire on the effect of wearing the HAL (Fig. 5B), 66.6% of caregivers answered positively to the question “Does the use of the HAL reduce physical burden (such as the burden on the lower back)?” In addition, 50% of caregivers answered positively to the question “Does the use of the HAL reduce mental burden?”

## Discussion

In this study, an observational time-motion analysis was used to identify the care tasks for which wearable transfer support robots were used in nursing facilities. The survey was conducted at nursing facilities in which MS or HAL had been in use on a daily basis for morse than 24 months. We confirmed common characteristics such as whether robots were utilized intensively in direct care situations, in particular, focusing on transfer assistance and toileting care, which are particularly burdensome on the lumbar region. Furthermore, the HAL was used not only for transfer assistance and toileting care, but also for meal assistance due to its compactness compared to the MS and its ability to be used in the sitting position. On the other hand, the MS was not suitable for meal assistance since sitting on a chair with the MS is difficult due to its size. Instead, the MS was used specifically in a routine manner for burdensome tasks in the early morning (mainly for toileting care and transfer assistance that occur around the bed of the care recipient) and that the care operation was designed to achieve these tasks. These results suggest the possibility of diversifying the care scenarios in which robots can be used by understanding their characteristics through long-term use. We believe that these observations provide useful information for the future introduction of robotic care equipment and for their sustainable and effective use within nursing facilities. Our results also showed that in both robots, about half of the transfer and toileting assistance were performed without the use of the robots (Table 1). In both nursing facilities, we heard that the time for wearing the robots was generally decided by a schedule to share the use of the robots among caregivers. Thus, this suggests that transfer and toileting assistance still occurred even outside the wearing time of the robots. It is hoped that the introduction of a larger number of robots in each nursing facility will have further benefits.

The use of the HAL for care support has been suggested to decrease lumbar load [23, 24] and decrease muscle activity in the lower back [13] during repetitive lifting tasks, suggesting its use has the potential to reduce the risk of lower back pain and improve lifting performance. Similarly, the use of the MS has been reported to reduce muscle activity in the lower back and may reduce local muscle fatigue [12, 25] when lifting heavy weights. However, these results were obtained from specific and limited movements in an experimental environment, and no study has examined what kind of work these robots can actually be used for when they are installed and incorporated into working operations in nursing homes. For the first time, this study used an observational time-motion approach to examine the characteristics of the care tasks and times at which robots are used in skilled facilities that have been utilizing wearable transfer support robots for more than 24 months.

In this study, a common characteristic of both robots was that they were worn specifically for direct care of transfer assistance and toileting care. Previous studies have indicated that a combination of lumbar-loading movements is required for caregivers to reposition a care recipient in bed, which can potentially cause disability depending on the recipient’s weight and motor function [4]. Repetition of this task further increases the risk of low back pain and associated disability [3, 26]. Conversely, toileting care in this study included undressing and dressing in the toilet, as well as tasks associated with changing diapers around the bed. According to these observations, the intensive use of the MS and HAL in transfer assistance and toileting care can be considered as an effort to reduce the burden in the tasks most attributable to load on the lumbar region. Furthermore, both robots were used intensively in the morning for toileting care. In interviews with the staff using the MS, they reported that care rounds for medication administration, toileting care, and transfer assistance were conducted in the mornings, along with wakeup calls for the care recipients, at which time the pre-assigned MS wearers use it as part of their “routine work.” In the interviews regarding the HAL, the staff reported that they try to wear the equipment in advance, anticipating that some tasks will be performed as “routine work,” because direct care that occurs unexpectedly may not be completed in time, considering the time it takes to put the equipment on, as well as other preparations. These results indicate that the robots may be more effective for long-term use with their incorporation into pre-determined regular care tasks, rather than tasks that occur unexpectedly.

In addition, bathing care is one of the most physically demanding tasks in the caregiving environment [11, 27]. Some use of the HAL was identified for bathing care, but both robots were only used for a limited amount of time for this task. The HAL was used for certain care recipients, but not used widely, because the equipment protrudes around the waist that could cause it to hit the walls of the bathroom. The MS was not used for assistance in the bathtub because an older version that was not waterproof was used in this study. In all cases, the use of these robots, based on an understanding of their characteristics, was noticeable in dressing/undressing and preparation in the room outside of the bathroom before and after bathing. An important finding will be how well the MS can be used for bathing care in the future when the equipment is replaced with the latest waterproof version.

The work survey of all staff members showed that the greatest amount of time was spent on meal assistance among the direct care tasks in both facilities. The importance of the time spent on meal assistance and staffing for meal assistance has been pointed out [28, 29]. However, when compared to transfer assistance, toileting care, and bathing care, the frequency of use of the robots for meal assistance was lower because fewer movements are required to move, transfer, or hold the care recipient. The purposeful use of the HAL was observed, in which, depending on the care recipient, the caregiver may need to maintain a forward leaning posture while seated when providing assistance with meals. Thus, the HAL, which can be worn while seated, is used to reduce physical burden. In addition, regarding the time of use throughout the day, use of the HAL was identified at various times during the afternoon. This may be due to the inclusion of “snacks” between meals, which take up much of the afternoon for care recipients who are unable to meet their caloric needs with only three meals [30, 31]. Conversely, it was suggested that the MS may not be suitable for meal assistance because users cannot be seated while wearing it.

One of the other tasks in this study was the maintenance of the robots, which has been pointed out as one of the challenges for introducing robotic care equipment due to the time and effort required. However, the amount of time spent on equipment maintenance per day was only approximately 5 min, suggesting that maintenance is not problematic in nursing facilities that have been using the robots for a long period of time. However, in addition to preparation and storage, the HAL also needs to be charged, and the nursing facility has implemented measures to efficiently manage charging by regularly charging the batteries at a fixed location and time on a daily basis in order to eliminate this problem as much as possible.

According to work surveys conducted in previous time-motion studies of nursing facilities, there are a wide variety of tasks such as direct care for care recipients (e.g., transfer assistance, toileting care, meal assistance, and bathing care) [16–18, 32–34] as well as medication administration [35–37] and documentation that are required for the safe management of care recipients [17]. A similar variety of tasks was identified in the present study, highlighting the amount of time and variety of tasks that caregivers are required to perform on a daily basis. Traditionally, the workload of caregivers reduces the amount of time they can spend on care recipients, and, as a result, care recipients do not engage in conversation or ask for whatever it is they need [31, 38]. A common characteristic of the two robots examined in this study was that the majority of staff members felt that their use for transfer assistance could reduce their physical burden. The results support the possibility that using these robots in the care settings may be useful for reducing physical burden on caregivers. Furthermore, if the various tasks identified by the observational time-motion analysis can be performed effectively through the use of the equipment, it will be possible not only to reduce the physical burden on staff members, but also to provide safer and more comfortable care. In the future, if the sustainable use of technology can reduce the physical burden on caregivers, it may also reduce their mental burden and provide a sense of security. If the use of such robots can create a more relaxed environment and more free time for the staff, it may help caregivers and care recipients to form closer relationships.

Finally, the present study has some limitations that should be discussed. First, although two different robots were selected as representative wearable care equipment for transfer assistance, there are many other types of assistive equipment that can be expected to reduce the burden on caregivers’ lower back [39–41]. Waters et al. recommended low-friction drawsheets or slider sheets to facilitate patient repositioning [42]. The use of ceiling lifts has also been effective in reducing the burden on caregivers and the frequency at which they suffer from disability [3]. However, it is expected that caregivers may be hesitant to use such equipment considering the limited locations in which they can be installed [43, 44]. In addition, mobile lifts may have the same effect, but the time required to attach the sling seat to a care recipient is a barrier for their use [45]. In any case, it will be possible to obtain information on the situations in which each type of equipment is used in the work setting and effective care operations by expanding the range of assistive equipment to be surveyed in the future. Second, although this study was conducted in facilities that have been using the robotic equipment continuously for at least 24 months, the process of learning to use the equipment in facilities that are introducing them for the first time should be examined. From the interviews in this study, it was found that, in both facilities, in addition to identifying the situations in which the robots were used, training sessions were held to facilitate staff members’ familiarity with the equipment, and innovations in care operations were practiced to ensure efficient use of the equipment. Eventually, it will be necessary to perform additional studies to increase the level of proficiency effectively based on the acquired information. In addition, a limitation of the observational time-motion analysis conducted in this study was its 1-min temporal resolution, which may have prevented the investigation of events that ended within 1 min. In the future, it will be necessary to work on the development of automatic job analysis and evaluation tools with higher temporal resolution by applying image recognition and other techniques using video cameras.

## Conclusion

In this study, observational time-motion analysis was carried out to identify the care scenarios in which wearable robotic care equipment is utilized in nursing facilities that have used the equipment on a daily basis for an extended period of time. In both facilities, the equipment was confirmed to be used intensively for tasks that place a high burden on the lower back. The sustained use of these robots could lead to a reduction in the risk of low back pain, which is an urgent issue for caregivers, and the associated decline in the turnover of the caregiving workforce. In other words, this will lead to the creation of an environment in which caregivers can work for a long period of time with a sense of security and will help to ease the chronic shortage of caregivers. These findings could be developed as useful information for the achievement of better care using robots in the future.

## Supplementary Information

**Additional file 1: Supplementary Table 1.** The codes used for the time-motion study. The codes were translated from Japanese into English based on a chart produced by the Survey of Elderly Care by the Japanese Ministry of Health, Labour, and Welfare in 2006 [21].

## Data Availability

The datasets analyzed during the current study are not publicly available given that the research team has not completed its analysis, but are available from the corresponding author on reasonable request.

## Abbreviations

MS: Muscle Suit
HAL: HAL®, Lumbar Type for Care Support, time study: observational time-motion study (analysis)
