# Supplementary material for: Identification of care tasks for the use of wearable transfer support robots – an observational study at nursing facilities using robots on a daily basis
Source: BMC Health Serv Res. 2021 Jul 5;21:652. doi: 10.1186/s12913-021-06639-2 (PMC8256590; doi:10.1186/s12913-021-06639-2)
Supplement: Supplementary file 1 — Additional file 1: Supplementary Table 1. The codes used for the time-motion study. The codes were translated from Japanese into English based on a chart produced by the Survey of Elderly Care by the Japanese Ministry of Health, Labour, and Welfare in 2006 [21]. [file 12913_2021_6639_MOESM1_ESM.pdf]

Supplementary information for

Identification of care tasks for the use of wearable transfer support robots – an observational study at nursing facilities using robots on a daily basis

Authors:

Kenji Kato*[1], Tatsuya Yoshimi[1], Shohei Tsuchimoto[1], Nobuaki Mizuguchi[1], Keita Aimoto[2], Naoki Itoh[2], Izumi Kondo[2,3].

Affiliations:

1, Laboratory for Clinical Evaluation with Robotics, Center of Assistive Robotics and Rehabilitation for Longevity and Good Health, National Center for Geriatrics and Gerontology

2, Department of Rehabilitation Medicine, National Center for Geriatrics and Gerontology

3, Center of Assistive Robotics and Rehabilitation for Longevity and Good Health, National Center for Geriatrics and Gerontology

7-430, Morioka, Obu, Aichi 474-8511, JAPAN, Tel. +81-562-46-2311

(*to whom correspondently addressed, e-mail: kk0724@ncgg.go.jp)

Supplementary Table 1. Code chart

Major category	Middle category	Code: subcategory	Classification
1 Bathing / keeping clean / conditioning / changing clothes	1 Bathing	111 Bathing preparation	Direct care
		112 Bathing: verbal action, assistance, watching, etc.	
		113 Bathing cleanup	
	2 Wiping and cleansing	120 Wiping and cleansing	
	3 Hair washing	130 Hair washing	
	4 Washbasin / hand washing	140 Washbasin / hand washing	
	5 Oral and ear care	150 Oral and ear care	
	6 Dealing with menstruation	160 Dealing with menstruation	
	7 Conditioning	170 Conditioning	
	8 Changing clothes	180 Changing clothes	
	9 Others	190 Others	
2 Moving / transfers / changing position	1 Movement within the facility	211 Preparation for movement within the facility	
		212 Movement within the facility: verbal action, assistance, watching, etc.	
		213 Movement within the facility	
	2 Transfers	221 Transfer preparation	
		222 Transfers: verbal action, assistance, watching, etc.	
		223 Transfer cleanup	
	3 Getting up	231 Preparation for getting up	
		232 Getting up: verbal action, assistance, watching, etc.	
		233 Getting up: cleaning up	
	4 Standing up	241 Preparation for standing up	
		242 Standing up, verbal action, assistance, watching, etc.	
		243 Standing up: cleaning up	
	5 Other postural changes	251 Preparation for other posture changes	
		252 Other postural changes: verbal action, assistance, watching, etc.	
		253 Other postural changes: cleaning up	
	6 Putting on and taking off assistance tools	261 Preparation for attaching / detaching assistance tools	
		262 Attaching/ detaching assistance tools: verbal action, assistance, watching, etc.	
		263 Attaching/ detaching assistance tools: cleaning up	
	9 Others	290 Others	
3 Meal assistance	1 Cooking	310 Cooking	
	2 Serving / lower serving	320 Serving / lower serving	
	3 Dishwashing and cleaning up dishes	330 Dishwashing and cleaning up dishes	
	4 Eating (meals / snacks)	340 Eating (meals / snacks)	
	5 Hydration	350 Hydration	
	9 Others	390 Other	
4 Toileting	1 Urination	411 Preparation for urination	
		412 Urination: verbal action, assistance, watching, etc.	
		413 Urination cleanup	
	2 Defecation	421 Defecation preparation	
		422 Defecation: verbal action, assistance, watching, etc.	
		423 Defecation cleanup	
	9 Others	490 Others	
5 Life support for independence	1 Laundry	510 Laundry	Indirect care
	2 Cleaning and garbage disposal	520 Cleaning and garbage disposal	
	3 Organizing	530 Organizing	
	4 Food management	540 Food management	
	5 Money management	550 Money management	
	6 Door locking, fire extinguishing, disaster prevention	560 Door locking, fire extinguishing, disaster prevention	
	7 Awakening and sleeping	570 Awakening sleeping	Communication
	8 Other daily life	580 Other daily life	
	590 Conversation including consultation, advice and guidance, and other communication		
	0 Others	500 Others	

Major category	Middle category	Code: subcategory	Classification
6 Social life support	1 Events, club activities	610 Events, club activities	Communication
	2 Telephone, FAX, E-mail, letter	620 Telephone, FAX, E-mail, Letter	
	3 Document creation	630 Document creation	
	4 Responding to visitors	640 Responding to visitors	
	5 Movement when going out	650 Moving when going out	
	6 Acts on the go	660 Acts on the go	
	7 Relationship with the community	670 Relationship with the community	
	8 Professional training / production activities	680 Professional training / production activities	
	9 Social life training	690 Social life training	
	0 Other	600 Other	
7 Behavioral problems	710 Response when behavioral problems occur		Others
	720 Preventive response to behavioral problems		
	730 Preventive training for behavioral problems		
	9 Others	790 Others	
8 Medical	1 Use of drugs	810 Use of drugs	Medical care
	820 Respiratory, circulatory, digestive, and urinary procedures		
	830 Exercise equipment / skin / eye / otolaryngology / dental and surgical procedures		
	4 Observation / measurement / inspection	840 Observation / measurement / inspection	
	5 Guidance / advice	850 Guidance / advice	
	6 Assistance when visiting a medical institution	860 Assistance when visiting a medical institution	
	9 Others	890 Others	
9 Physical function training	1 General	910 General	
	2 Applied daily life training	920 Applied daily life training	
	930 Speech / hearing training (speech / hearing therapy)		
	4 Sports training	940 Sports training	
	5 Traction / heat / electrotherapy	950 Traction / heat / electrotherapy	
	9 Others	990 Others	
0 Care that is not directly related to the recipient	How about to use service like "1 Service about the recipient"	011 Contact / coordination	Indirect care
		012 Recording / document creation	Documentation
		013 Residents' units, floors, etc., environment maintenance and cleaning	Indirect care
		014 Resident goods management (including goods purchasing)	
		015 Patrol and overlooking using installed equipment	
		016 Patrol, overlooking (other than that)	Transit
	2 Service about staff	021 Hand washing	Indirect care
		022 Waiting (napping)	Staff Break
		023 Records and coordination regarding staff	Documentation
		024 Break	Staff Break
		025 Environmental maintenance and cleaning for staff	Indirect care
		026 Moving	Transit
		027 Maintenance of installed equipment	Equipment
		028 About staff (other)	Indirect care
	9 Others	099 Others	
10 Correspond with other units		999 Correspond with other units	Others

Code chart	1 Bathing / keeping clean / conditioning / changing clothes	Direct care
	2 Moving / transfers / changing position	
	3 Meal assistance	
	4 Toileting	
	5 Life support for independence	Indirect care/ Communication
	6 Social life support	Communication
	7 Behavioral problems	Others
	8 Medical	Medical care
	9 Physical function training	
	0 Business that is not directly related to the resident	Various
	10 Correspond with other units	Others
